# Metagenomic Profile of Bacterial Communities of *Hyalomma scupense* and *Hyalomma asiaticum* Ticks in Kazakhstan

**DOI:** 10.3390/pathogens14101008

**Published:** 2025-10-06

**Authors:** Kulyaisan T. Sultankulova, Nurlan S. Kozhabergenov, Gaukhar O. Shynybekova, Meirim D. Almezhanova, Samat B. Zhaksylyk, Madina R. Abayeva, Olga V. Chervyakova, Takhmina O. Argimbayeva, Mukhit B. Orynbayev

**Affiliations:** 1 Research Institute for Biological Safety Problems, National Holding QazBioPharm, The Ministry of Healthcare of the Republic of Kazakhstan, Kordai District, Zhambyl Region, Gvardeiskiy 080409, Kazakhstan; 2 Research and Production Center MVA Group, Koksay Village, Karasai District, Almaty 040921, Kazakhstan; 3 National Academy of Sciences of Kazakhstan under the President of the Republic of Kazakhstan, Almaty 050010, Kazakhstan

**Keywords:** tick, *H. scupense*, *H. asiaticum*, microbiome, bacteria, sequencing, 16S rRNA

## Abstract

Ticks are important vectors of pathogens affecting humans and animals, posing a serious threat to health. For the first time, we studied the metagenomic profile of the microbial composition of *Hyalomma scupense* and *Hyalomma asiaticum* ticks in Kazakhstan. A total of 94 adult *H. asiaticum* and *H. scupense* ticks collected from randomly selected cattle in Kazakhstan in 2023 were analyzed. 16S rRNA gene sequencing was performed using the Ion Torrent NGS platform. Taxonomic classification was carried out in the BV-BRC platform with the Kraken2 database. Metagenomic analysis revealed 26 bacterial genera, including both pathogenic and symbiotic taxa. In *H. scupense*, the dominant groups were *Francisella* (89.0%), *Staphylococcus* (76.0%) and *Candidatus Midichloria* (61.0%), while in *H. asiaticum,* they were *Francisella* (99.0% and 95.0%) and *Helcococcus* (65.0%). In male *H. scupense*, the proportion of *Francisella* reached 89%, whereas in females, it varied from 2% to 28%. In *H. asiaticum*, *Helcococcus* accounted for 65% in males compared to 11% in females. This is the first report on the metagenomic profile of the microbiota of *H. scupense* and *H. asiaticum* in Kazakhstan. The detection of pathogens indicates a risk of their transmission to humans and animals and highlights the need to develop new tick control strategies.

## 1. Introduction

Ticks are widely distributed across the globe, and tick infestations pose a significant threat to livestock due to their capacity to transmit tick-borne pathogens (TBPs) and cause various diseases. Ticks are considered the second most important vectors of human diseases worldwide after mosquitoes, yet they are the primary vectors of pathogenic diseases in both domestic and wild animals [1,2]. In addition to pathogens, ticks also harbor a diverse array of symbiotic and commensal microorganisms [3,4].

Currently, 896 tick species have been recorded worldwide, of which 27 species belong to the genus *Hyalomma* [5,6]. The tick fauna of Kazakhstan comprises over 30 ixodid tick species, including 8 species of the genus *Hyalomma.* The natural conditions of Kazakhstan are favorable for the habitation of various tick species, among which *H. scupense* and *H. asiaticum* are prominent. In the desert landscapes of Kazakhstan, *H. asiaticum* predominates, whereas *H. scupense* is more common in semi-desert and low-mountain steppe regions. Ticks from the southern region of Kazakhstan are recognized as vectors and reservoirs of multiple pathogens, causing Q fever, tick-borne spotted fevers, arboviruses, and piroplasmoses [7,8,9,10,11,12].

Recent studies have shown that *Hyalomma* ticks serve as reservoirs and vectors for a wide range of pathogens, including bacteria and novel RNA viruses found in ticks [13,14,15]. These ticks pose a dual threat to both human health and agriculture, highlighting the need for ongoing epidemiological monitoring. Current data analysis reveals that the metagenomic profile of bacterial communities in *H. scupense* and *H. asiaticum* ticks inhabiting the southern and southeastern regions of Kazakhstan remains insufficiently studied.

Furthermore, the potential applications and prospects of metagenomic approaches in the diagnosis and epidemiological surveillance of infectious diseases have not yet been considered. Until recently, most studies focused primarily on the identification of tick-borne pathogens and their epidemiology using traditional methods [9,10,11,12]. Advancements in metagenomics have fundamentally transformed the ability to characterize the taxonomic composition of microbial ecosystems [16]. Recent metagenomic projects have revealed that the diversity of life is far greater and more complex than previously imagined through classical methods that rely on visually observable biodiversity [17,18,19,20].

To date, there are no available data on the microbiome of the most widespread and significant *Hyalomma* tick species in Kazakhstan. In this research, 16S rRNA metagenomics was employed to investigate bacterial communities in ticks collected from cattle in the southern and southeastern regions of Kazakhstan.

The use of the 16S rRNA metagenomic sequencing method was chosen because of its versatility, high sensitivity, and ability to detect bacteria that cannot be identified through traditional culture methods [21]. It is especially useful for identifying both dominant and rare taxa, including potential symbionts and pathogens [22]. Due to its high sensitivity, reproducibility, and relative accessibility, 16S rRNA metagenomics is an ideal tool for comprehensive analysis of tick microbiota [3]. In Kazakhstan, *H. asiaticum* and *H. scupense* are of significant epidemiological and veterinary importance [23]. They are primary vectors of particularly dangerous pathogens and reservoirs for various bacterial and viral agents. These tick species are widespread in the southern region of the country, making them key targets for epidemiological surveillance and control [24]. Therefore, this study aimed to investigate and compare the bacterial microbiome of *H. asiaticum* and *H. scupense* collected from cattle in southern Kazakhstan using 16S rRNA gene sequencing. The findings of this work may contribute to disease risk assessment for livestock and human populations in these areas and serve as a foundation for developing targeted control strategies.

## 2. Materials and Methods

### 2.1. Tick Sampling

Tick collection was conducted in April–September 2023 in the Kyzylorda, Zhambyl, Turkestan, and Zhetysu regions of Kazakhstan. All ticks were collected from cattle. The collection was performed in accordance with the permit issued by the Committee for Veterinary Control and Supervision of the Ministry of Agriculture of the Republic of Kazakhstan and with the consent of the animal owners. During tick collection, personnel adhered to strict safety measures, including wearing protective suits with sealed collars and cuffs, and regularly performing self- and mutual inspections to detect any crawling or attached ticks.

Ticks were removed using blunt forceps from the inner thigh, udder, scrotum, neck, and axillary regions of the animals. Live ticks were placed into plastic tubes with screw caps. To maintain humidity, a leaf of a cereal plant was usually added to each tube. Prior to analysis, ticks were kept alive in a cool place or refrigerated in standard tubes with plant material. Detailed labels were attached to all collected samples. Each arthropod was identified using an Altami PC0745 stereomicroscope (RS0745, Altami, Saint Petersburg, Russia). A total of 1260 ticks were collected. For subsequent microbiome analysis, 94 adult *H. asiaticum* and *H. scupense* ticks were randomly selected from cattle across various locations in the southern and southeastern regions of Kazakhstan (Table 1). Female tick samples were pooled in groups of 10 based on location, species, and sex. Two separate pools of male ticks were created, consisting of 6 and 8 individuals, respectively. Additional information, including collection sites and tick counts, is provided in Table 1.

**Figure 1:**
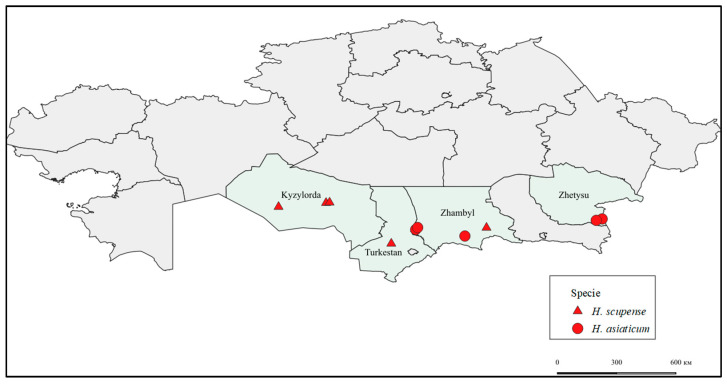
Visualization of the geographic distribution of H. scupense and H. asiaticum ticks using cartographic analysis in QGIS. 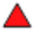
—*Kyzylorda_Zhalagash1_H. scupense*, *Kyzylorda_Zhalagash2_H. scupense*, *Kyzylorda_Kazaly_H. scupense*, *Zhambyl_Shu_H. scupense*, *Turkestan_Otyrar_H. scupense*. 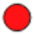
—*Zhambyl_Zhanatas1_H. asiaticum, Zhambyl_Zhanatas2_H. asiaticum*, *Zhambyl_Ryskulov_H. asiaticum*, *Zhetysu_Zharkent_H. asiaticum*, *Zhetysu_Chulakai_H. asiaticum*.

Geographic distribution of *H. scupense* and *H. asiaticum* ticks visualized using cartographic analysis performed in QGIS software version 3.34. (Figure 1).

### 2.2. Molecular-Genetic Identification of Ticks

Tick specimens were initially identified using morphological methods [25], using an Altami stereomicroscope (RS0745, Altami, Saint Petersburg, Russia). The morphological identifications were subsequently confirmed by molecular genetic analysis targeting the mitochondrial cytochrome c oxidase subunit 1 gene (COX1) [26].

A fragment of the COX1 gene (820 bp) was amplified by polymerase chain reaction (PCR) for molecular identification using the following primers: Cox1F: 5′-GGAACAATATATTTAATTTTTGG-3′ and Cox1R: 5′-ATCTATCCCTACTGTAAATATATG-3′. The amplification conditions were as follows: initial denaturation at 94 °C for 2 min; followed by 35 cycles of denaturation at 94 °C for 30 s, annealing at 54 °C for 45 s, and extension at 72 °C for 1 min; with a final extension step at 72 °C for 10 min [27].

PCR products of the COX1 gene were subjected to nucleotide sequencing on an Applied Biosystems 3130 automated DNA sequencer (ABI, 3130, Foster City, CA, USA) using the Bigdye Terminator V3.1 loop sequencing kit (ABI, Vilnius, Lithuania). The obtained nucleotide sequences were analyzed using the Sequencher v. 4.5 program (Gene Codes Corporation, Ann Arbor, MI, USA). The nucleotide sequence was aligned using the Mega 7.0 computer program complex. A set of nucleotide sequences from the international GenBank database of the National Center for Biotechnology Information (NCBI) was used to construct a phylogenetic tree. Phylogenetic analysis of the sequences was performed using the Mega 11.0 program.

For the analysis, COX1 gene sequences of ticks obtained from GenBank were used (accession numbers: MW498400, JQ737073, OR533789, NC053941, MN907845, MN821375, MN964348, MN907831, OM743222.

### 2.3. DNA Extraction

Ticks, previously sterilized with 70% ethanol, were homogenized using a mechanical homogenizer in centrifuge tubes containing 500 µL of chilled sterile phosphate-buffered saline (PBS, 1×). The homogenized samples were then centrifuged at 12,000× *g* for 10 min at 4 °C, and the supernatant was collected. Total DNA was extracted from the supernatant using the QIAamp DNA Mini Kit (Qiagen, Hilden, Germany) following the manufacturer’s protocol. The purity of the extracted DNA was assessed by agarose gel electrophoresis, and DNA samples were stored at −80 °C until further use.

### 2.4. Library Preparation

DNA concentration from the microbial community was measured using the Qubit™ dsDNA HS (High Sensitivity) Assay Kit (Life Technologies, Carlsbad, CA, USA). Library preparation was carried out using the Ion 16S™ Metagenomics Kit (Thermo Fisher Scientific, Waltham, MA, USA). Briefly, 12 µL of DNA was mixed with 15 µL of Environmental Master Mix. Subsequently, 3 µL of each 10× 16S Primer Set was added: one tube with primers targeting regions V2–4–8 (Pool 1) and another with primers targeting V3–6, 7–9 (Pool 2). Samples were subjected to PCR under the following thermal cycling conditions: initial denaturation at 95 °C for 10 min; followed by 25 cycles of 95 °C for 30 s, 58 °C for 30 s, and 72 °C for 30 s; with a final extension at 72 °C for 7 min. Amplification products were purified using AMPure XP beads (Beckman Coulter, Brea, CA, USA) and eluted in nuclease-free water. The concentrations of PCR products from Pool 1 and Pool 2 were assessed by agarose gel electrophoresis and subsequently combined.

End repair was performed by adding 20 µL of 5× End Repair Buffer and 1 µL of End Repair Enzyme to each sample, followed by incubation at room temperature for 20 min. The pooled amplicons were purified again using AMPure XP beads and eluted in Low TE buffer. Ligation and nick repair were performed using 10× Ligase Buffer, Ion P1 Adaptor, Ion Xpress™ Barcodes, dNTP Mix, DNA Ligase, Nick Repair Polymerase, nuclease-free water, and sample DNA. The thermal protocol included incubation at 25 °C for 15 min followed by 72 °C for 5 min. Adapter-ligated and nick-repaired DNA was again purified using AMPure XP beads and eluted in Low TE buffer.

Library amplification was performed using the Ion Plus Fragment Library Kit (Thermo Fisher Scientific, Carlsbad, CA, USA) under the following PCR conditions: 95 °C for 5 min; followed by 7 cycles of 95 °C for 15 s, 58 °C for 15 s, and 70 °C for 1 min; and a final extension at 70 °C for 1 min. The amplified library was purified using AMPure XP beads and eluted in Low TE buffer. The optimal library concentration for template preparation was quantified by qPCR on a QuantStudio™ 5 Real-Time PCR System (Applied Biosystems, Foster City, CA, USA) using the Ion Universal Library Quantitation Kit (Thermo Fisher Scientific, Waltham, MA, USA). Each library was normalized to a concentration of 30 pM, and equal volumes of each library were pooled for downstream processing.

### 2.5. Sequencing

Libraries were prepared for sequencing using the Ion Chef Instrument and the Ion 510™ & 520™ & Ion 530™ Kit–Chef (Thermo Fisher Scientific, Waltham, MA, USA). Chips were then loaded onto the Ion GeneStudio S5 System (Ion Torrent platform) (Thermo Fisher Scientific, Marsiling Industrial Estate, Woodlands, Singapore) along with Ion S5 Sequencing Kit reagents (Thermo Fisher Scientific, Waltham, MA, USA) and sequenced at the laboratory. Samples in this study were sequenced on Ion 530 chips using 400 bp sequencing size.

### 2.6. Taxonomic Classification

Taxonomic analysis of the bacterial community was performed by high-throughput sequencing of the hypervariable region V2–4–8 and V3–6, 7–9 of the 16S rRNA gene on the Ion Torrent platform using next-generation sequencing technology. The obtained data were taxonomically classified on the platform of The Bacterial and Viral Bioinformatics Research Center (BV-BRC) using the standard Kraken2 database.

### 2.7. Statistical Data Analysis

Alpha diversity parameters of microbial communities were assessed by calculating the Shannon-Wiener diversity index [28], Simpson’s dominance index [29], and Margalef’s richness index [30].

Beta diversity analysis to compare the taxonomic composition of the microbiomes of *H. scupense* and *H. asiaticum* ticks was performed using the Bray–Curtis dissimilarity index [31]. To quantify the similarity of bacterial community species composition between *H. scupense* and *H. asiaticum*, the Jaccard index was applied [32]. Table 2 presents the formulas used for the quantitative estimation of alpha and beta diversity parameters.

All analyses were performed and processed using the R programming environment (https://www.r-project.org/). To analyze the geographical distribution of bacteria associated with *H. scupense* and *H. asiaticum*, Principal Coordinates Analysis (PCoA) was employed. Data visualization was carried out using the Python v3.13.5 programming language. Data visualization was also conducted in Python. Hierarchical clustering analysis was applied to identify natural groupings of bacteria and tick samples based on their microbiome profiles. The clustering method used was agglomerative hierarchical clustering with the nearest neighbor (single linkage) algorithm.

## 3. Results

### 3.1. Tick Collection and Identification

Tick samples randomly selected for microbiome analysis were identified to the species level using a combination of morphological and molecular data.

**Figure 2:**
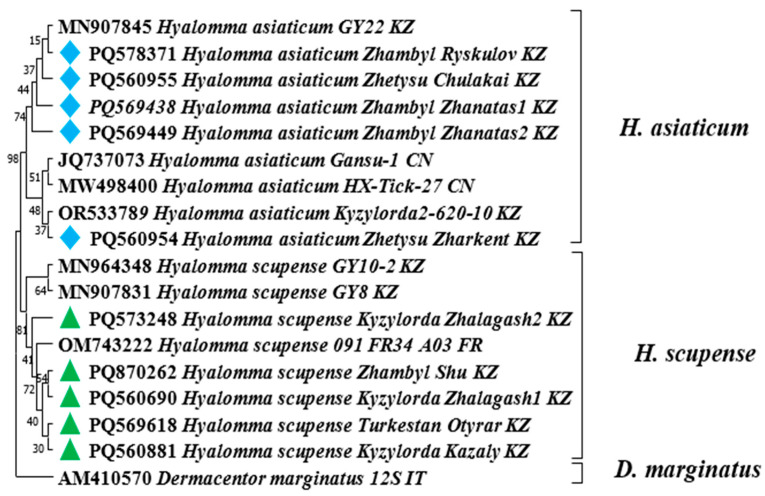
Phylogenetic analysis of the cytochrome c oxidase subunit I (COX1) gene of *H. scupense* and *H. asiaticum* ticks. The phylogenetic tree was constructed by the maximum likelihood method using the Tamura–Nei model for nucleotide sequences in MEGA 11. 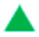
—*Hyalomma scupense* identified in this study based on the COX1 gene. 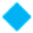
—*Hyalomma asiaticum* identified in this study based on the COX1 gene.

A total of 94 adult ticks, initially morphologically identified and grouped into 10 pools, were subjected to PCR amplification targeting a fragment of the cytochrome c oxidase subunit I (COX1) gene, followed by sequencing of PCR products from positive samples. Phylogenetic analysis of the COX1 gene sequences confirmed the morphology-based identification of the ticks as *H. scupense* and *H. asiaticum*. The COX1 gene sequences of *H. scupense* obtained in this study have been deposited in GenBank under the following accession numbers: PQ560690 (*H. scupense Kyzylorda_Zhalagash1_KZ*), PQ560881 (*H. scupense Kyzylorda_Kazaly_KZ*), PQ573248 (*H. scupense Kyzylorda_Zhalagash2_KZ*), PQ569618 (*H. scupense Turkestan_Otyrar_KZ*), and PQ870262 (H. scupense Zhambyl_Shu_KZ). The COX1 gene sequences of *H. asiaticum* have been deposited in GenBank under accession numbers: PQ569438 (*H. asiaticum Zhambyl_Zhanatas1_KZ*), PQ569449 (*H. asiaticum Zhambyl_Zhanatas2_KZ*), PQ560954 (*H. asiaticum Zhetysu_Zharkent_KZ*), PQ560955 (*H. asiaticum Zhetysu_Chulakai_KZ*), and PQ578371 (*H. asiaticum Zhambyl_Ryskulov_KZ*) (Figure 2).

### 3.2. Assessment of the Bacterial Diversity Profile Based on 16S rRNA Gene Sequencing

**Figure 3:**
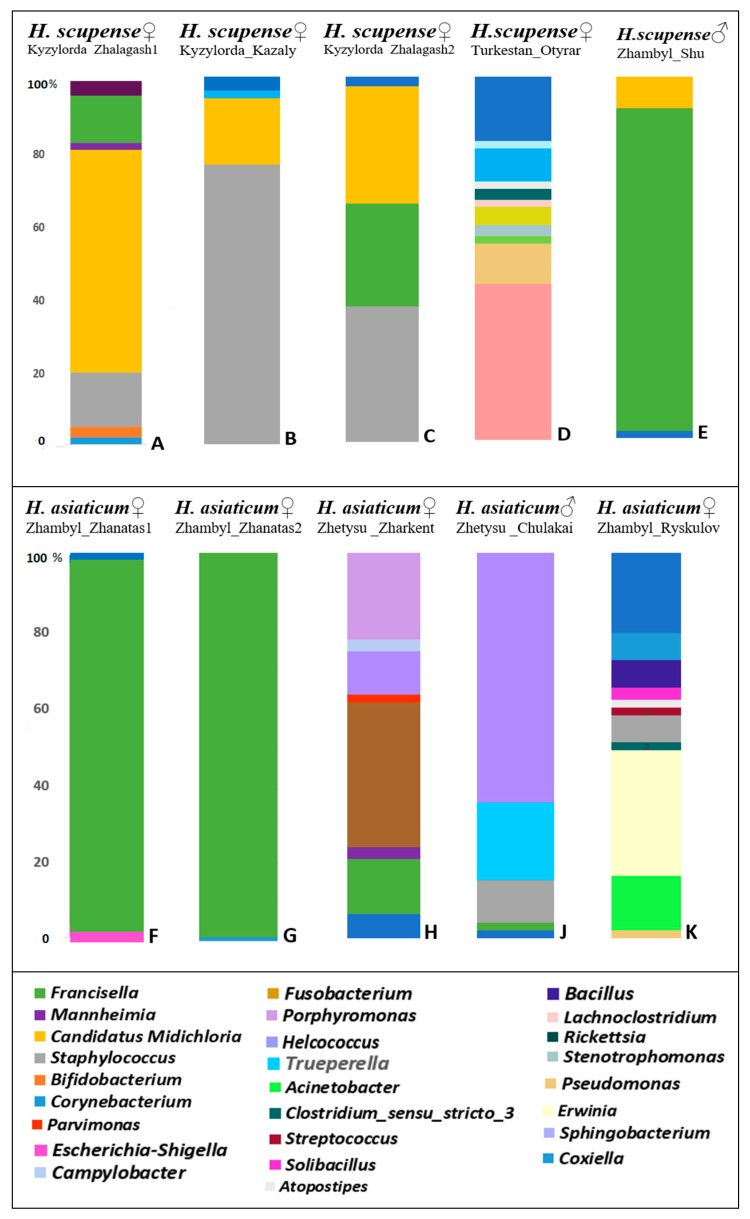
Metagenomic profiles of the prevalence of 26 major bacterial genera detected in *H. scupense* and *H. asiaticum* tick samples using the Ion GeneStudio S5 System.

All 10 tick pools were analyzed for the presence of tick-borne bacterial agents using 16S rRNA gene sequencing data processed on the Ion Torrent next-generation sequencing platform. The raw reads are available at NCBI SRA under BioProject PRJNA1305121: SRR35607863, SRR35607862, SRR35607858, SRR35607856, SRR35607855, SRR35607854, SRR35607853, SRR35607861, SRR35628833, and SRR35628829. Analysis of the bacterial composition of *Hyalomma* ticks collected from cattle in different regions of Kazakhstan demonstrated clear differences between *H. scupense* and *H. asiaticum* (Table 3, Figure 3).

At the genus level, the bacterial community composition in *H. scupense* was dominated by *Francisella* (89.0%), *Staphylococcus* (76.0%), and *Candidatus Midichloria* (61.0%), whereas in *H. asiaticum*, a higher relative abundance of *Francisella* (99.0% and 95.0%) and *Helcococcus* (65.0%) was observed.

The results indicate that the microbiome of Kazakhstani ticks varies in composition and microbial diversity depending on the geographic region. From a geographic perspective, *H. scupense* and *H. asiaticum* ticks collected from the Zhambyl region exhibited significantly higher prevalence of *Francisella* (99% in Zhambyl_Zhanatas2, 95% in Zhambyl_Zhanatas1, and 89% in Zhambyl_Shu) compared to other locations.

*H. scupense* ticks from southern regions of Kazakhstan (Turkestan, Zhambyl, and Kyzylorda regions) showed a higher relative abundance of *Candidatus Midichloria* (61% in Kyzylorda_Zhalagash1), whereas this microorganism was not detected in *H. asiaticum* or *H. scupense* from the Zhetysu region.

*H. scupense* from the Turkestan region exhibited elevated relative abundances of several bacterial genera, including *Coxiella* (43.0%), *Pseudomonas* (11.0%), *Acinetobacter* (2.0%), *Stenotrophomonas* (3.0%), *Rickettsia* (5.0%), *Lachnoclostridium* (2.0%), *Clostridium sensu stricto* 3 (3.0%), *Atopostipes* (2.0%), *Corynebacterium* (9.0%), and *Sphingobacterium* (2.0%) (Figure 3D).

*H. asiaticum* from the Zhambyl region harbored more abundant bacterial genera such as *Pseudomonas* (3.0%), *Acinetobacter* (14.0%), *Erwinia* (32.0%), *Clostridium sensu stricto 3* (2.0%), *Staphylococcus* (7.0%), *Streptococcus* (2.0%), *Atopostipes* (2.0%), *Solibacillus* (3.0%), *Bacillus* (7.0%), and *Corynebacterium* (7.0%) (Figure 3K).

The endosymbiont *Francisella* constituted a significantly dominant proportion of the microbiome in male *H. scupense* ticks (89.0%, Figure 3E) compared to females (13.0%, Figure 3A; 28%, Figure 3C; and 2%, Figure 3D).

Similarly, *Helcococcus* comprised a significantly dominant percentage of the microbiome in male *H. asiaticum* ticks (65.0%, Figure 3J) compared to females (11.0%, Figure 3H).

### 3.3. Bacterial Microbiome Diversity in H. scupense and H. asiaticum

**Figure 4:**
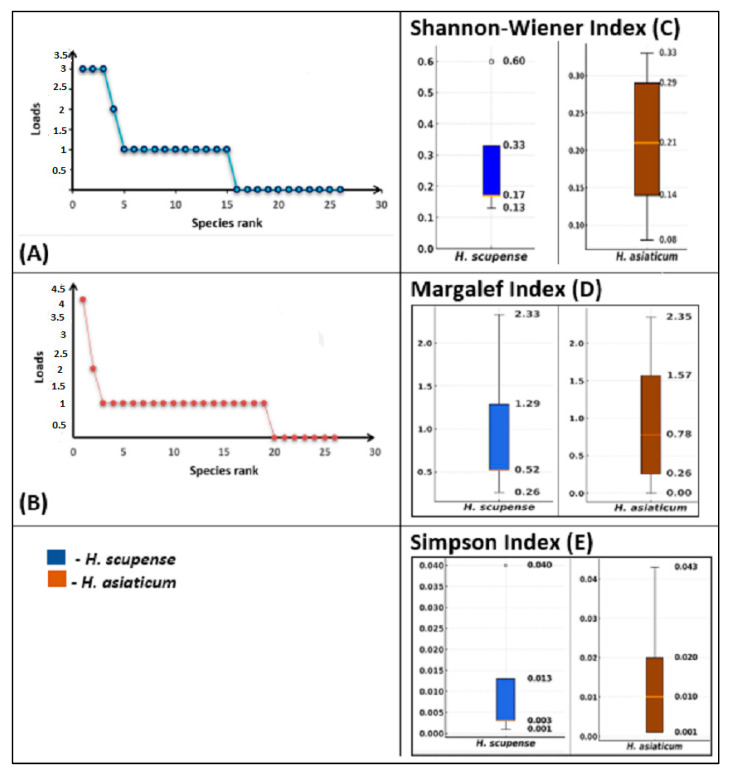
Assessment of alpha diversity and variation in bacterial communities in *H. scupense* and *H. asiaticum* ticks. Rank and abundance diagrams of bacterial communities in *H. scupense* (**A**) and *H. asiaticum* (**B**). Shannon-Wiener index (**C**), Margalef index (**D**), and Simpson index (**E**). The line inside each boxplot indicates the median value.

Alpha diversity of bacterial communities associated with *H. scupense* and *H. asiaticum* ticks is determined by the Shannon-Wiener, Margalef, and Simpson indices (Figure 4).

The bacterial abundance distribution curves for *H. scupense* (Figure 4A) and *H. asiaticum* (Figure 4B) show that a small number of bacterial genera (top ranks) carry the greatest abundance, while the majority of taxa occur at lower abundance levels. The broken shape of the curve suggests the presence of dominant bacteria with a gradual decline in abundance among other taxa. Notably, *H. asiaticum* demonstrates a more pronounced dominance of a single bacterial genus, whereas *H. scupense* exhibits a more even distribution among several top-ranked genera.

Shannon-Wiener index (Figure 4C): the median for *H. scupense* was 0.17 (interquartile ranges (IQR) 0.17–0.33), for *H. asiaticum*–0.21 (IQR 0.14–0.29), which reflects the high uniformity of communities in *H. asiaticum*.

Margalef index (Figure 4D): for *H. scupense* the median is 0.52 (IQR 0.52–1.29), *for H. asiaticum* the median is—0.78 (IQR 0.26–1.57), which indicates species richness in *H. asiaticum.*

Simpson index (Figure 4E): median values were 0.003 (IQR 0.003–0.013) for *H. scupense* and 0.010 (IQR 0.001–0.020) for *H. asiaticum*, confirming the high diversity in *H. asiaticum.*

### 3.4. Microbial Variations Depending on Geographic Origin

We constructed distribution diagrams and cladograms of microbial communities, demonstrating differences in the bacterial composition of *H. scupense* and *H. asiaticum* ticks according to their geographic origin. To assess differences in bacterial communities between regions, principal coordinate analysis (PCoA) was performed. Coordinates were calculated based on the Bray–Curtis distance matrix.

The microbiome of *H. asiaticum* showed no significant differences between regions (*p*-value = 0.299, *p* > 0.05, ANOVA test), suggesting a similar bacterial composition across different locations. This indicates that *H. asiaticum* harbors a relatively stable microbiota independent of geographic location.

**Figure 5:**
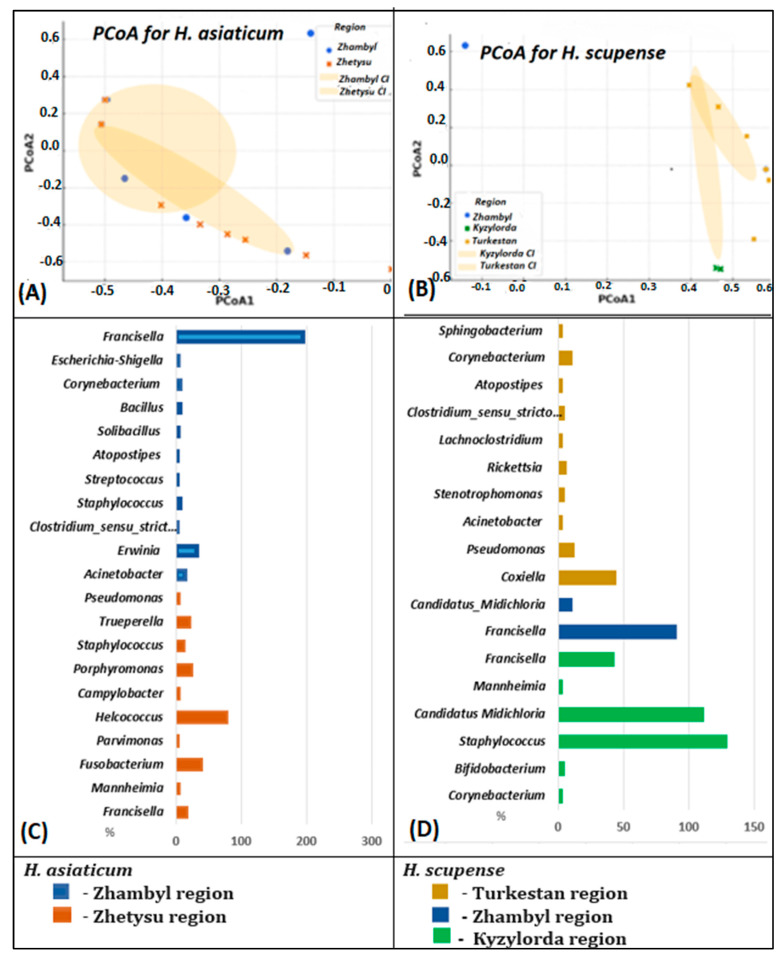
Geographic distribution of bacteria carried by *H. scupense* and *H. asiaticum* ticks based on Principal Coordinates Analysis (PCoA). Scatter plots visualize the data with PCoA1 and PCoA2 axes. Each point on the graphs corresponds to tick samples of *H. asiaticum* (**A**) and *H. scupense* (**B**), with point colors indicating their regional origin. Microbial community cladograms for *H. asiaticum* (**C**) and *H. scupense* (**D**) illustrate bacterial composition differences according to regions of Kazakhstan: Kyzylorda, Turkestan, Zhambyl and Zhetysu.

For *H. scupense*, differences between regions were statistically significant, with a *p*-value of 0.023 (*p* < 0.05) according to the ANOVA test. The microbiome of *H. scupense* significantly varies among regions, which is supported by the visual analysis of the PCoA plot. A statistically significant difference in bacterial composition was observed between the Kyzylorda and Zhetysu regions for *H. scupense* (*p* = 0.016). This may indicate that environmental or host-related factors differ between these regions. For example, the climate in the Kyzylorda region is more arid and located further south, whereas the Zhetysu region is more humid and situated to the east, potentially leading to distinct microbial community structures (Figure 5).

### 3.5. Beta Diversity of the Bacterial Community in the Microbiome of H. scupense and H. asiaticum Ticks

**Figure 6:**
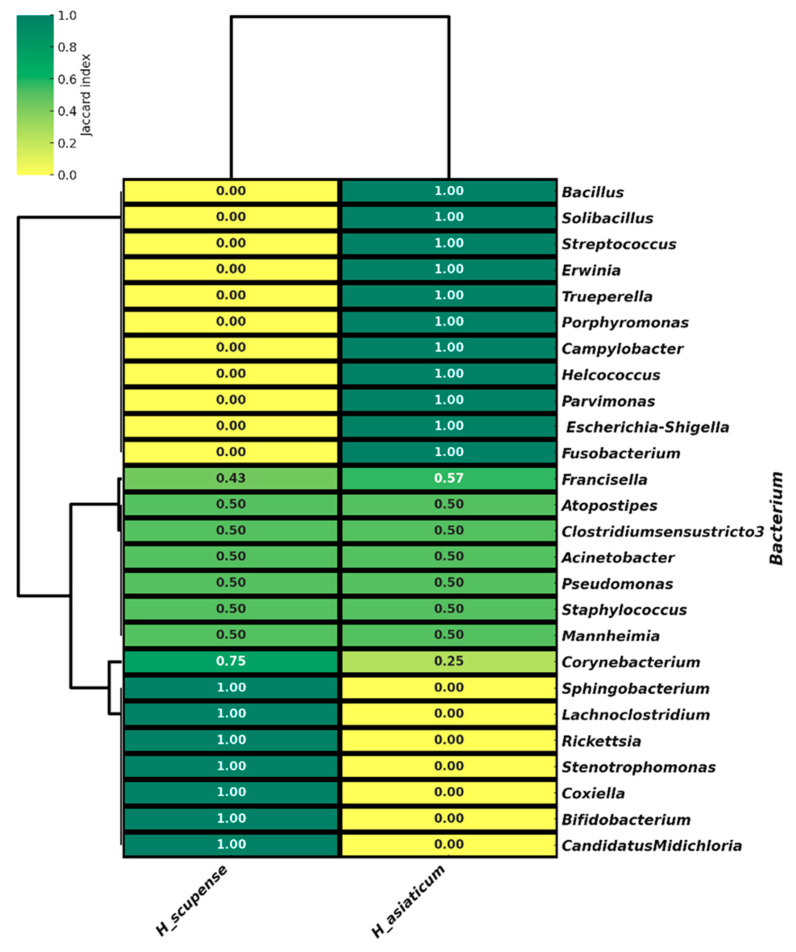
Heatmap of the relative abundance of bacteria present in *H. scupense* and *H. asiaticum* ticks. Light yellow shades correspond to low similarity (Jaccard index ≤ 0.50), while dark green shades indicate high similarity (Jaccard index > 0.50). The heatmap was generated using the Python environnt. The clustering method applied was agglomerative nearest neighbor clustering.

The Jaccard index was used to assess the similarity in bacterial community composition between *H. scupense* and *H. asiaticum*, reflecting the proportion of shared bacterial taxa between sample pairs. Hierarchical clustering analysis was applied to identify natural groups of bacteria within the tick microbiomes (Figure 6).

The constructed heatmap with hierarchical clustering revealed patterns in the distribution of bacterial composition among ticks. Statistically significant differences between groups (*p* < 0.001, *t*-test) indicate that bacterial communities of different tick species form distinguishable clusters. Some bacterial genera, such as *Mannheimia*, *Staphylococcus*, *Pseudomonas*, *Acinetobacter*, *Clostridium sensu stricto 3*, and *Atopostipes*, are present in both samples with equal proportions (0.5), indicating partial overlap in bacterial composition. The genera *Corynebacterium* (0.75 and 0.25) and *Francisella* (0.43 and 0.57) occur in both samples but at different frequencies. The similarity level of bacterial communities between *H. scupense* and *H. asiaticum* ticks based on the Jaccard index is only 30.8%, with only 8 shared bacterial genera identified.

Certain bacteria, including *Coxiella*, *Candidatus Midichloria*, *Stenotrophomonas*, *Rickettsia*, *Lachnoclostridium*, *Sphingobacterium*, and *Bifidobacterium*, are found exclusively in *H. scupense*, indicating a species-specific bacterial composition. Conversely, genera such as *Fusobacterium*, *Escherichia-Shigella*, *Parvimonas*, *Helcococcus*, *Campylobacter*, *Porphyromonas*, *Trueperella*, *Erwinia*, *Streptococcus*, *Solibacillus*, and *Bacillus* are unique to *H. asiaticum*, reflecting bacterial diversity specific to this tick species.

Thus, the analysis demonstrates that the bacterial communities of the two tick species comprise both shared and unique taxa, suggesting differences in their ecology or potential interactions with pathogens.

## 4. Discussion

Ticks of the genus *Hyalomma* are one of the most widespread and epidemiologically significant genera of ixodid ticks, found primarily in the steppe, semi-desert, and desert regions of Eurasia, the Middle East, and Africa [33,34,35]. These ticks are well adapted to hot and arid climates, are frequently found in pasture areas, and are closely associated with farm animals, primarily cattle and small ruminants, as well as camels and horses. Due to their high ecological plasticity and ability to migrate long distances with their hosts, *Hyalomma* play a key role in maintaining natural foci of vector-borne infections and pose a serious threat to human and animal health [14,15].

Studies of *Hyalomma* microbial populations not only expand knowledge about their biology and ecology, but also have direct practical implications for the epidemiology of transmissible infections [36]. Studying the microbiota allows us to better understand potential pathogen transmission routes, identify factors influencing the formation of the bacterial community, and develop new approaches to biosecurity and the fight against tick-borne infections [20]. In Kazakhstan, there is very little data on the composition of microbial communities in the most common species—*H. scupense* and *H. asiaticum*. Studying their microbiota and symbionts is important for understanding the bacterial communities of ticks. Such research can help to elucidate their ability to transmit pathogens to vertebrate hosts.

In the bacterial microbiota of Kazakhstan ticks *H. scupense* and *H. asiaticum*, the symbiotic/pathogenic bacterium *Francisella* predominates in the microbiome. According to literature data, *Francisella* predominates in the microbiome of *H. excavatum* and *H. marginatum* [37].

Recent studies confirm the widespread distribution of *Francisella* in other representatives of the genus *Hyalomma. Francisella*-like endosymbionts are transmitted vertically [38] and constitute the main components of the *Hyalomma* microbiome worldwide [20]. Thus, they have been identified in *H. anatolicum* in Pakistan, *H. lusitanicum* in Spain, *H. aegyptium* in Turkey, *H. dromedarii* in the Middle East, and also in *H. asiaticum* in China [17,39,40,41].

These results demonstrate that the *Francisella* symbiont/pathogen plays a key role in shaping the microbial communities of *Hyalomma* ticks and likely has a significant impact on its vector competence. Bacterial endosymbionts may influence tick physiology and reproductive capacity, as well as the ability of ticks to transmit transmitted pathogens, and finally may interact with tick hosts, which may have veterinary and zoonotic implications, in particular for *Francisella* and *Rickettsia* bacteria [15]. *Staphylococcus* and *Helcococcus* were the most common pathogens in *H. scupense* and *H. asiaticum*, respectively, indicating the possible presence of these microorganisms in animal populations parasitized by these tick species.

Identification of *Helcococcus* spp. is challenging due to the slow growth of these organisms [42]. In our study, *Helcococcus* was identified using 16S rRNA gene sequencing. Members of the genus *Helcococcus* are known to cause mastitis and urocystitis in animals [43]. Different species of *Staphylococci* vary in their virulence, which determines different levels of health threat. *Staphylococcus* probably enters the tick’s body from the environment and persists throughout its life cycle [44].

Their exact role is still unknown, but pathogenic species such as *Staphylococcus lentus* and *Staphylococcus saprophyticus*, which were previously found in *Hyalomma* ticks [45], may be the reason why staphylococci are frequently detected in these ticks. In our study, Staphylococcus accounted for 7.0% of the microbiota in *H. asiaticum* and 76% in *H. scupense*, confirming the prevalence of this genus of bacteria in these tick species. *Candidatus Midichloria mitochondrii* is a widespread endosymbiont of ticks [46]. This bacterium plays a critical role in the growth and development of the host, providing it with additional sources of ATP and B vitamins, which contributes to an increase in the adaptive capabilities and general biology of ticks [47]. Previously, *Candidatus Midichloria mitochondrii* was detected in *H. anatolicum* in Xinjiang, China [48]. In our study, this endosymbiont was detected only in *H. scupense*.

Blood feeding is known to have a strong impact on tick microbial diversity, composition, and species richness [49]. Ticks acquire more microorganisms, including pathogens, from hosts during blood feeding. More diverse and larger numbers of vertebrate hosts result in a more diverse microbiota [50].

Despite the presence of a common bacterial base, a significant proportion of the bacterial composition differs between *H. scupense* and *H. asiaticum*. The data obtained highlight both the species-specificity of microbial associations and the potential influence of environmental [51,52], geographic [53] and other factors on the formation of microbial communities in ticks.

Our study revealed a difference in the microbiota composition between the sexes of *H. scupense* and *H. asiaticum* ticks. In male *H. scupense*, a higher prevalence of the endosymbiont *Francisella* was found, which constituted the dominant part of the microbiome—89% in samples from Zhambyl_Shu. At the same time, in females, the proportion of *Francisella* was significantly lower: 13%—Kyzylorda_Zhalagash 1, 28% Kyzylorda_Zhalagash 2 and only 2%—Turkestan_Otyrar. This difference may indicate sexual characteristics in the formation of microbial communities and the role of endosymbionts in *H. scupense*, which requires further study.

*H. asiaticum* exhibits significant sex differences in the microbiota composition. In males from the Zhetysu_Chulakai region, the proportion of *Helcococcus* was 65%, while in females from Zhetysu_Zharkent it was only 11%. This indicates a potential sex specificity in the formation of the bacterial community. According to the literature, *Helcococcus* was not so widely detected in *H. truncatum* collected from cattle; it was present with a relative frequency of about 4.7% [54].

Males and females may have different contacts with the host, habitat, and food, which may determine differences in the microbiota, including the predominance of *Helcococcus* in males.

Our results expand our understanding of the biodiversity and microbiota ecology of *Hyalomma* in Central Asia and highlight the need for further research to understand the contribution of endosymbionts and pathogens to the vector competence of these ticks. These results are particularly important for Central Asian countries, where *Hyalomma* ticks are widespread and play an important role in the transmission of zoonotic infections. The lack of data on the microbiota of local tick populations hinders understanding of epidemiological risks in the region. Our results fill this gap and provide a basis for the development of preventive and diagnostic strategies aimed at reducing the threat to human and animal health in Kazakhstan and neighboring Central Asian countries.

Cattle play a key role in food security, being the main source of meat and milk, making them an important target for assessing the risk of transmission of tick-borne pathogens [55]. Our study is limited to using ticks collected only from cattle. We chose this approach because cattle are of great epidemiological importance in Kazakhstan. Furthermore, collecting ticks from only one animal species allowed us to conduct sampling under identical conditions, making the results more comparable. However, this limitation may have biased the results, as it did not account for the influence of other hosts and the environment on the tick microbiome. Including ticks collected from wild animals or vegetation would be a promising avenue for future research.

## 5. Conclusions

Our study characterized the microbial communities of *H. scupense* and *H. asiaticum* from the southern and southeastern regions of Kazakhstan for the first time using next-generation sequencing.

*H. scupense* and *H. asiaticum* ticks contained 15 and 19 genera of bacteria, respectively, with only eight (30.8%) being common. 69.2% of the bacterial composition consisted of species-specific taxa, with 26.9% for *H. scupense* and 42.3% for *H. asiaticum.* The dominance of *Francisella* in both species confirms its key role in the microbiome of the genus *Hyalomma*, while the identified sex differences—the predominance of *Francisella* in male *H. scupense* and *Helcococcus* in male *H. asiaticum*—indicate an additional level of regulation of microbial associations. These results expand our understanding of the biodiversity and microbiota ecology of *Hyalomma* in Central Asia and highlight the need for further research aimed at understanding the contribution of endosymbionts and pathogens to the vector competence of these ticks.

## Figures and Tables

**Table 1:** Tick sampling information from southern and southeastern regions of Kazakhstan.

	Sample Collection Site	Coordinates	No. of Ticks	Tick Species	Sex	Host	Genbank Accession No
1	Kyzylorda_Zhalagash1	45°04′55″ 64°40′51″	10	*H. scupense*	♀	Cattle	PQ560690
2	Zhambyl_Zhanatas1	43°34′00″ 69°45′00″	10	*H. asiaticum*	♀	Cattle	PQ569438
3	Kyzylorda_Kazaly	44°51′00″ 61°59′24″	10	*H. scupense*	♀	Cattle	PQ560881
4	Kyzylorda_Zhalagash2	45°04′55″ 64°40′51″	10	*H. scupense*	♀	Cattle	PQ573248
5	Zhambyl_Zhanatas2	43°34′00″ 69°45′00″	10	*H. asiaticum*	♀	Cattle	PQ569449
6	Zhambyl_Shu	43°40′32″ 73°45′40″	8	*H. scupense*	♂	Cattle	PQ571834
7	Zhetysu_Zharkent	44°10′00″ 80°00′00″	10	*H. asiaticum*	♀	Cattle	PQ560954
8	Zhetysu_Chulakai	44°05′15″ 79°58′00″	6	*H. asiaticum*	♂	Cattle	PQ560955
9	Zhambyl_Ryskulov	43°12′00″ 72°32′00″	10	*H. asiaticum*	♀	Cattle	PQ578371
10	Turkestan_Otyrar	42°46′36″ 68°22′09″	10	*H. scupense*	♀	Cattle	PQ569618

**Table 2:** Indices for quantitative assessment of alpha and beta diversity parameters.

Index Name	Formula	Diversity Level
Shannon–Wiener diversity index	$H^{\prime }=-\sum_{i=1}^{R}\left(p_{i}lnp_{i}\right)$	Alpha diversity
Margalef richness index	$d=\frac{S-1}{lnN}$	Alpha diversity
Simpson’s dominance index	D= ∑$\left(\frac{n_{i}\left(n_{i}-1\right)}{N\left(N-1\right)}\right)$	Alpha diversity
Bray–Curtis dissimilarity index	$K_{B-C}=\frac{2\sum Nmin}{N_{a}+N_{b}}$	Beta diversity
Jaccard similarity index	$I_{J}=\frac{a}{a+b+c}$	Beta diversity

Note: Shannon-Wiener index (H′): where pipi is the proportion of individuals belonging to the ii-th species, RR is the total number of species, and ln denotes the natural logarithm. Margalef’s index (d): where SS is species richness (total number of species), and NN is the sample size (total number of individuals in the community). Simpson’s index (D): where nini is the number of individuals of the ii-th species, and NN is the total number of individuals in the sample. Bray–Curtis dissimilarity index (BC): where NaNa is the total abundance (sum of counts) in the first community, NbNb is the total abundance in the second community, and Nmin represents the sum of the lesser abundances for each taxon shared between the two communities. Jaccard similarity index (J): where aa is the number of species common to both lists, bb is the number of species present only in the first list, and cc is the number of species present only in the second list.

**Table 3:** Content (%) of bacteria identified in *H. scupense* and *H. asiaticum* ticks collected from cattle in various regions of Kazakhstan.

	Tick Sample	HSKZ1, %	HSKK, %	HSKZ2, %	HSTO, %	HSZS, %	HAZZ1, %	HAZZ2, %	HAZZ, %	HAZC, %	HAZR, %
Genus of Bacteria											
*Corynebacterium*		2	2	-	9	-	-	-	-	-	7
*Bifidobacterium*		3		-	-	-	-	-	-	-	-
*Staphylococcus*		15	76	37	-	-	-	-	-	11	6
*Candidatus Midichloria*		61	17	32	-	9	-	-	-	-	-
*Mannheimia*		2	-	-	-		-	-	3	-	-
*Francisella*		13	-	28	-	89	95	99	14	2	-
*Coxiella*		-	-	-	43	-	-	-		-	-
*Pseudomonas*		-	-	-	11	-	-	-	37	-	-
*Acinetobacter*		-	-	-	2	-	-	-	-	-	14
*Stenotrophomonas*		-	-	-	3	-	-	-	-	-	2
*Rickettsia*		-	-	-	5	-	-	-	-	-	-
*Lachnoclostridium*		-	-	-	2	-	-	-	-	-	-
*Clostridium sensu stricto 3*		-	-	-	3	-	-	-	-	-	2
*Atopostipes*		-	-	-	2	-	-	-	-	-	2
*Sphingobacterium*		-	-	-	2	-	-	-	-	-	-
*Escherichia-Shigella*		-	-	-	-	-	3	-	-	-	-
*Fusobacterium*		-	-	-	-	-	-	-	-	-	-
*Parvimonas*		-	-	-	-	-	-	-	2	-	-
*Helcococcus*		-	-	-	-	-	-	-	11	65	-
*Campylobacter*		-	-	-	-	-	-	-	3	-	-
*Porphyromonas*		-	-	-	-	-	-	-	23	-	-
*Trueperella*		-	-	-	-	-	-	-	-	20	-
*Erwinia*		-	-	-	-	-	-	-	-	-	32
*Streptococcus*		-	-	-	-	-	-	-	-	-	-
*Bacillus*		-	-	-	-	-	-	-	-	-	7
*Solibacillus*		-	-	-	-		-	-	-	-	7
Other		4	5	3	18	2	2	1	7	2	21
1. *H. scupense Kyzylorda_Zhalagash1*—HSKZ1; 2. *H. scupense Kyzylorda_Kazaly*—HSKK; 3. *H. scupense Kyzylorda_Zhalagash2*—HSKZ2; 4. *H. scupense Turkestan_Otyrar*—HSTO; 5. *H. scupense Zhambyl_Shu*—HSZS; 6. *H. asiaticum Zhambyl_Zhanatas1*—HAZZ1; 7. *H. asiaticum Zhambyl_Zhanatas2*—HAZZ2; 8. *H. asiaticum Zhetysu _Zharkent*—HAZZ; 9. *H. asiaticum Zhetysu _Chulakai*—HAZC; 10. *H. asiaticum Zhambyl_Ryskulov*—HAZR.											

## Data Availability

The data are available in the article. Further inquiries can be directed to the corresponding authors.
